# Recreational Exposure to Low Concentrations of Microcystins During an Algal Bloom in a Small Lake

**DOI:** 10.3390/md20080018

**Published:** 2008-06-26

**Authors:** Lorraine C. Backer, Wayne Carmichael, Barbara Kirkpatrick, Christopher Williams, Mitch Irvin, Yue Zhou, Trisha B. Johnson, Kate Nierenberg, Vincent R. Hill, Stephanie M. Kieszak, Yung-Sung Cheng

**Affiliations:** 1 National Center for Environmental Health, Centers for Disease Control and Prevention, 4770 Buford Highway NE, Chamblee, Georgia 30341, USA; 2 Department of Biological Sciences, Wright State University, 3640 Colonel Glen Highway, Dayton, Ohio 45435. Current address: 42184 Tweedle Lane, Seaside, Oregon 97138, USA; 3 Mote Marine Laboratory, 1600 Ken Thompson Parkway, Sarasota, Florida 34236, USA; 4 Greenwater Laboratories, 205 Zaegler Drive, Pakatka, Florida 32177, USA; 5 Lovelace Respiratory Research Institute, Inhalation Toxicology Laboratory, P.O. Box 5890, Albuquerque, New Mexico 87285, USA; 6 National Center for Zoonotic, Vector-Borne, and Enteric Diseases, Centers for Disease Control and Prevention, 4770 Buford Highway NE, Chamblee, Georgia 30341, USA; 7 Atlanta Research and Education Foundation, 1670 Clairmont Road, Decatur, Georgia 30033, USA

**Keywords:** Aerosol exposures, blue-green algae, cyanobacteria, microcystins, Microcystis aeruginosa, waterborne exposures

## Abstract

We measured microcystins in blood from people at risk for swallowing water or inhaling spray while swimming, water skiing, jet skiing, or boating during an algal bloom. We monitored water samples from a small lake as a *Microcystis aeruginosa* bloom developed. We recruited 97 people planning recreational activities in that lake and seven others who volunteered to recreate in a nearby bloom-free lake. We conducted our field study within a week of finding a 10-μg/L microcystin concentration. We analyzed water, air, and human blood samples for water quality, potential human pathogens, algal taxonomy, and microcystin concentrations. We interviewed study participants for demographic and current health symptom information. Water samples were assayed for potential respiratory viruses (adenoviruses and enteroviruses), but none were detected. We did find low concentrations of *Escherichia coli*, indicating fecal contamination. We found low levels of microcystins (2 μg/L to 5 μg/L) in the water and (<0.1 ng/m^3^) in the aerosol samples. Blood levels of microcystins for all participants were below the limit of detection (0.147μg/L). Given this low exposure level, study participants reported no symptom increases following recreational exposure to microcystins. This is the first study to report that water-based recreational activities can expose people to very low concentrations of aerosol-borne microcystins; we recently conducted another field study to assess exposures to higher concentrations of these algal toxins.

## Introduction

For hundreds of years, harmful algal blooms (HABs) and HAB-related toxins have been associated with incidents of wild and domestic animal poisonings and outbreaks of human illnesses [1, 2, 3, 4]. In documented incidents, illnesses were typically associated with eating seafood contaminated with marine HAB toxins, drinking water contaminated with cyanobacterial (blue-green algal) HAB toxins, or with failures to protect water used for medical purposes, such as kidney dialysis [5, 6].

In addition to consuming toxins directly in contaminated food or water, people are, during outdoor recreational or occupational activities, exposed to HAB-related toxins in their environment. For example, recent work has shown that brevetoxins produced by *Karenia brevis*, the organism responsible for Florida red tides, can be incorporated into marine aerosols and blown onshore [7]. When these aerosols are inhaled, they induce respiratory symptoms in beach visitors and in healthy lifeguards [8, 9] and detectable changes in pulmonary function in people with asthma [10, 11].

Stewart *et al.* [12] reviewed case reports and anecdotal references that describe illnesses associated with exposure to cyanobacteria and related toxins during recreational activities in freshwater environments. The reported symptoms included hay-fever-like symptoms, gastroenteritis, pyritic skin rashes, and allergic reactions. Others, however, reported more severe acute reactions including severe headaches, fever, and blistering in the mouth. One anecdotal report (John Burns, personal communication, 2001) suggested that people exposed to aerosols on golf courses and lawns watered with local pond water experience respiratory irritation. In addition to these case reports and anecdotes, epidemiologic studies examining recreational exposures to cyanobacteria have been published [13, 14, 15, 16, 17]. Most studies did not find an association between recreational exposure to cyanobacteria and adverse health effects. However, Pilotto *et al.* [17] did find a significant trend of increasing symptom reports with longer water contact time and exposure to higher cyanobacteria counts. In addition, people in this study who were exposed for more than 1 hour to water with low concentrations of cyanobacteria (> 5,000 cells per ml) were more likely to complain of at least one symptom during the 7 days following exposure than were people who were exposed to waters that did not contain cyanobacteria (odds ratio = 3.44; 95% confidence interval (CI), 1.09 – 10.82).

More recently, Stewart *et al.* [18] conducted a study of over 3,500 people who used personal water craft on small freshwater lakes in southeast Queensland (Australia), coastal New South Wales, or Florida (US). In the follow-up phone interview conducted at least 3 days following the water-contact activities, people who used personal watercraft on lakes with high levels of cyanobacteria (cell surface area >12.0 mm^2^/mL) were 2.1 (CI, 1.1 – 4.0) times more likely to report symptoms—particularly respiratory symptoms—than were people who used their personal watercraft on lakes with low levels of cyanobacteria (cell surface area < 2.4 mm^2^/mL).

Using available evidence that supports an association between exposure to waters containing high concentrations of cyanobacteria and subsequent adverse health effects, many entities, including the World Health Organization (WHO), Australia, and some other European countries, have developed guidelines for managing recreational waters with cyanobacterial blooms [19, 20]. The WHO guidelines, for example, are based on the following three tiers of potential public health threat: 1) low probability of adverse health effects from water with ≤ 20,000 cells/mL or 10 μg chlorophyll-a/L where cyanobacteria predominate, 2) moderate probability of adverse health effects from waters with 100,000 cells/mL or 50 μg chlorophyll-a/L, and 3) high probability of adverse health effects from contact with, ingestion, or inhalation of cyanobacteria when algal scum appears on the water surface. These guidelines are based on cell concentrations rather than on cyanobacteria toxin concentrations. Because a given bloom may or not produce toxins, it is possible that the guidelines may be too conservative in terms of limiting access to recreational waters. Epidemiologic studies to assess symptoms and to measure concentrations of cyanobacterial toxins in the environment (water and aerosols) could provide data needed to develop more specific recreational exposure guidelines.

Our primary objective was to assess whether we could measure microcystins in blood from people at risk for swallowing water or inhaling spray while engaged in water-related recreational activities (e.g., water skiing, tubing, swimming, and using personal watercraft) in a lake during a microcystin-producing algal bloom. In addition, we were interested in determining whether microcystin concentrations in aerosol and water samples were associated with microcystin concentrations in blood and with self-reported symptoms.

## Results and discussion

### Study site

We were aware of a number of small recreational lakes in Michigan, New York, Ohio, and other states that historically experienced microcystin-producing algal blooms in late summer, and we corresponded with colleagues conducting routine monitoring of these lakes. Based on previous experience with accidental exposures to water contaminated with microcystins, we expected that people involved in activities that involve ingesting water (i.e., swimming) or inhaling aerosols (i.e., jet-skiing, water skiing, or sailing a small boat) should receive enough exposure to allow us to detect microcystins in their blood. The minimum amount of microcystins detectable in blood was about 0. 9 ng microcystins in 10 ml of blood (approximately 0.1 ppb). This was very close to the 1 ng limit of detection for the ELISA assay.

Accordingly, we planned to conduct the field study within a week of receiving monitoring results that the microcystin concentrations in one of the lakes was at least 10 μg/L. Delays in conducting the tests and unpredictable changes in local weather can, however, rapidly change algal bloom characteristics. For this field study, water sample analyses completed after the study found that even with a significant, visible bloom of organisms capable of producing microcystins, the concentrations of microcystins were actually in the range of 2–5 ug/L. Thus people may have been exposed, but the waterborne microcystin concentrations were not high enough to detect microcystin in blood samples from our study subjects. Nevertheless, in this field study we verified that inhalation is a possible route of exposure to microcystins, and these data can be compared with data from studies that used the same protocol in lakes with potentially higher exposures.

#### Water Samples

We collected 24 water samples (four sample sites, each sampled in the morning and afternoon, on each of the 3 study days, August 4–6, 2006) for analysis of water quality, alga taxa, and microcystin concentrations from the lake with the algal bloom. Table 1 contains the ranges of water quality parameters tested over the 3-day study period as well as weather data collected during that same period.

In general, cyanobacterial populations are found and thrive in warm, calm waters with high concentrations of nutrients and characterized by consistent, relatively high pH and dissolved oxygen (DO) levels. High-pH (8–10) and DO (above saturation) would be a result of relatively high population densities and are not a prerequisite for growth. Still, if potentially toxigenic species were present, warm temperatures (25–30° C), low wind action (0–3 mph), and high nutrient loads (phosphorus and nitrogen), together with clear sunny skies, would stimulate growth and increase the potential risk for toxin production.

Several factors contributed to the weak bloom and low concentrations of microcystins in the water we tested. First, microcystins are endotoxins stored and isolated inside algal cells, thus they do not readily diffuse or leak into the aquatic environment. Under normal environmental conditions in a healthy, reproductively active toxin-producing bloom event, 95% to 98% of the toxins would be intracellular. Accordingly, we would expect to find toxin only in water containing cells. Second, during the sampling period for the lake with the bloom, winds generally increased in speed from morning to afternoon, thus disorganizing bloom accumulations and diluting microcystin concentrations by scattering algal cells. Third, *Microcystis* species are buoyant, surface-floating algae that tend to accumulate on or near shore as winds blow them landward. Three of the four samples sites where water samples were collected were open water—thus we would not expect high toxin concentrations unless the bloom was extensive. For example, early one morning before the winds had increased in strength, we sampled a small accumulation of cells and found microcystin concentrations of 43 ug/L. Later that morning and in the afternoon we did not observe cell accumulations, and in the water samples, toxin concentrations remained relatively low (3 ug/L – 5 ug/L). In addition, during the study period a storm with significant winds (> 5 mph) further dispersed the bloom.

#### Algal taxonomy

We assessed algal taxonomy in 12 samples collected during the morning of each of the 3 study days from the exposure lake at the four sampling stations. The range of phytoplankton concentrations was 175,000 cells/mL to 688,000 cells/mL. Over 95% of the cells were cyanobacteria.

Table 2 contains the densities of potentially toxigenic cyanobacteria (PTOX-C). During the sampling period, densities ranged from 54,000 total cells/mL to 144,000 total cells/mL. The dominant genera of PTOX-C reported in water samples were *Anabaena*, *Aphanizomenon*, *Cylindrospermopsis*, and *Microcystis*. The two documented microcystin-producing genera present were *Anabaena* and *Microcystis*. No *Planktothrix* species were observed. The dominant species present were *Anabaena macrospora*, *Aphanizomenon aphanizomenoides/gracile*, and *Microcystis cf. botrys. Aphanizomenon aphanizomenonoides/gracile* is, however, a suspected microcystin producer. Because the toxin levels were very low, we could not examine in the collected water samples any potential relationship between the number of microcystin-producing PTOX-C and the concentrations of microcystins.

#### Air Samples

On each of the three study days we detected microcystins at levels near the level of detection (LOD) (0.0037 ng/m^3^) on each stage of the high volume, airborne particle impactor. The flow rate, sampling time, and total concentration of microcystins from the five stages and the back-up filters, respectively, were as follows: day 1, 1.17 m^3^/min, 360 min, 0.050 ng/m^3^; day 2, 1.15 m^3^/min, 386 min, 23 ng/m^3^; and day 3, 1.22 m^3^/min, 484 min, 0.057 ng/m^3^.

We deployed 10 personal air samplers on study days 1 and 2, and 4 personal air samplers on study day 3. A summary of the mass and air concentration of microcystins as determined on each study day using the personal samplers is presented in Table 3. We were able to detect microcystins in 9 of 10 samples collected on day 1 and 6 of 9 samples on day 2. On day 3 we did not collect measurable concentrations of microcystins using personal air samplers.

#### Waterborne pathogens

Stewart *et al.* [18] found an increase in respiratory symptoms in people exposed to higher levels of cyanobacteria (surface area > 12.0 mm^2^/mL) than in people exposed to lower levels of cyanobacteria (surface area < 2.4 mm^2^/mL). It is possible that respiratory symptoms could be caused by infections from viruses rather than cyanobacteria. Thus, for our study, we added the analysis of the presence of adenoviruses, as well as enteroviruses in the lake water.

Each 10-L water sample was tested for adenoviruses using real-time PCR and enteroviruses using real-time reverse-transcription (RT)-PCR. Adenoviruses and enteroviruses were not detected in any water sample. Based on published detection limits for these assays, the testing protocol used in this study resulted in detection limits of approximately 1,250 adenovirus gene equivalent copies (GEC) per 10-L water sample and 200 enterovirus plaque-forming units (PFU) per 10-L sample. Thus, these data indicate that adenoviruses and enteroviruses were not present at substantial concentrations in the lake with the *Microcystis* bloom. In some samples, however, PCR inhibition was observed based on the delays in CT values measured for water samples seeded with an inhibitor standard versus CT values for seeded reagent-grade water controls. The range of CT value differences between seeded samples and controls was 0.6 to 9.3, with an average difference of 2.8 ± 2.0 CT values. This inhibition was likely due to the presence of bloom-associated constituents in the water samples—such as algal matter and other organic material (including humic and fulvic acids)—that are generally reflected by the relatively high turbidity data reported in Table 1.

The positive real-time PCR tests for *Escherichia coli* (data not shown), indicated that the organism was present at low levels (10 colony-forming units [cfu]/mL to 100 cfu/100 mL) in water from the lake with the bloom. *E. coli* is an established, fecal-specific water quality indicator that has been shown to correlate with reports of gastrointestinal illness in people exposed to the water during recreational activities [21, 22]. Thus despite the absence of any fecal-associated virus contamination in the lake with the algal bloom, we did find evidence of fecal contamination (i.e., the presence of *E. coli*).

#### Study participants

We recruited 104 study participants from lake visitors planning recreational activities that would generate aerosols, such as boating and using personal watercraft. Ninety-seven participants planned to use the lake with the bloom (the exposed group) and 7 planned to use a nearby lake with no bloom (the unexposed group). We obtained complete questionnaire data from 96 people in the exposed group and 7 in the unexposed group.

For the exposed group, the age range was 12 years to 67 years. Ninety-three (97%) were white, and 4 (4%) were of another race—no Hispanics were in the study. Forty-five (47%) of the exposed participants were female. Of the 96 exposed participants, fifty-seven (59%) reported that during the 7 days before the study they had participated in water-related recreational activities on the study lake. In addition, these exposed participants reported that in the year before the study, 75 (77%) had boated on, 34 (35%) had fished in, and 72 (74%) had swum in the study lake. Two (2%) reported using dietary supplements containing blue-green algae.

Study participants in the exposed group reported the following activities during the study period: swimming (73 [76%]), boating (34 [51%]), tubing (20 [30%]), riding personal water craft (13 [14%]), wake-boarding (8 [12%]), wading (3 [4%]), and fishing (2 [3%]). Of the study participants who swam, the amount of time ranged from 5 min to 180 min. Of those who rode personal watercraft, the amount of time ranged from 15 min to 120 min. Of those who did the other activities, the amount of time ranged from 10 min to 300 min Sixty-one (64%) of the exposed group reported that during the study period they had put their head underwater, and 39 (41%) reported they had swallowed water.

For the unexposed group, the age range was from 15 years to 58 years. All of the unexposed participants were white and non-Hispanic, and 4 (57%) were female. Of the 7 unexposed participants, 6 (86%) reported they participated in water-related recreational activities on the study lake (exposed site) during the 7 days before the study and in the year before the study. No one reported using dietary supplements containing blue-green algae.

The seven participants in the unexposed group reported only swimming during the study period. Six (86%) were in the water for 60 min, and one (14%) was in the water for 90 min. Six (86%) reported that during the study period they had put their head underwater, and 1 (14%) reported swallowing water.

Table 4 contains a summary of symptoms reported by both exposed and unexposed participants during the interviews. As a group, study participants reported more symptoms in the 7 days before the study than during study or during the 7 to 10 days after the study. In addition, as a group, study participants tended to report more symptoms (e.g., cough, sore throat for the exposed group, skin complaints for the unexposed group) immediately before doing study activities than immediately after such activities. A single participant in the control group reported five skin-related symptoms before going into the water but not after coming out of the water. No differences appeared in the frequency of reported symptoms between the exposed and control groups during the 7 days before the study, immediately before or immediately after doing study activities, or during the follow-up period.

We hypothesized that exposure to aerosolized microcystins would result in acute dermal and respiratory symptoms; thus, we expected people to report more symptoms immediately after study activities than during the week before the study. However, more people reported symptoms before doing study activities than after. In addition, participants reported more symptoms for the period of 7 days before the study than either immediately before or after doing study activities. Also, more than half of the participants in the exposed group and all but one of the participants in the control group had participated in water-related recreational activities on the study lake in the week before the study. Finally, the number of people reporting symptoms during the seven to 10 days after the exposure increased to levels that were more consistent with the number of people reporting symptoms for the period of 7 days before the study than with the number of people reporting symptoms either immediately before or immediately after doing study activities. While these differences in symptom reporting are not significantly different, the trend suggests that exposure to aerosolized microcystins may result in symptoms with onset a day or few days after the exposure, i.e., during the week after weekend activities on the blooming lake. This possibility could be addressed in a future study by identifying a study population that uses a lake that historically develops a summer bloom of microcystin-producing cyanobacteria and conducting symptom surveys before their first visit to the lake and then periodically throughout the summer.

#### Blood samples

We collected usable blood samples from 96 exposed and 6 unexposed participants. Of the 102 blood samples, 101 samples tested below the limit of detection according to the limit of detection described in the Envirologix Kit (0.147 μg/L). Only one blood sample showed detectable levels of microcystins—about 1.0 μg/L. LC/MS analysis of this sample showed the absence of microcystin-LR, -RR, and -YR. Given the LC/MS results, we concluded that this sample represented either a false positive or exposure from microcystin(s) not identified as microcystin-LR, -RR, or -YR. We did not identify anything unusual about this particular study participant (e.g., they did not use blue-green algae food supplements).

## Experimental Section

### Study approval

This study was approved by the Institutional Review Board of the Centers for Disease Control and Prevention.

### Environmental sampling sites

We used four sampling sites located along the major axis of a lake that had historical water quality data previously collected by Grand Valley State University. Each time sampling was performed latitude and longitude coordinates for all sample stations were recorded using a geographical positioning system (Magellan Map 330X, Magellan, Santa Clara, CA).

#### Water and plankton collection

Water samples were collected twice each day (AM and PM) at each sampling station for the identification of the algae present. Algal samples were collected at a depth of 0.15 meters. Water samples used for algal identification were immediately preserved with 10% Lugols solution and stored under dark, cool conditions.

Water samples were collected for microcystin analyses at a depth of 0.15 meters by directly immersing sample containers in ambient water. Before collection all sample bottles were rinsed with ambient water. In general, 4 liters per sample site were collected. To ensure that algae in samples remained alive and at low metabolic states until processing or preservation, collection bottles were stored in coolers under cool conditions (i.e., ice packs). To maintain algal health, bottles were also properly vented. After collection, all samples were stored chilled and mailed to GreenWater Laboratories/CyanoLab via overnight mail the day after the study was completed.

For algal identification and microcystin analyses, general water quality parameters (e.g., water temperature, dissolved oxygen, pH, specific conductivity, salinity, and turbidity) were measured at all sample sites twice per day at the same time as collection of the water samples. General water quality was measured by using a HACH multi-parameter data sonde (Hacj Company, Loveland, CO) supplied by Dr. R. Rediske of Grand Valley State University. Water quality parameters were measured at a depth of 0.15 meters. At the time of water collection ambient environmental conditions (cloud cover, wind speed, wind direction, and air temperature) were recorded at each site. Grand Valley State University performed water chemistry on the morning of August 4^th^, 2006.

#### Raw water sample preservation and algal taxonomy analysis

Samples were mixed to distribute evenly the phytoplankton cells. A 100-mL aliquot was removed and poured into a 125-mL amber polyethylene bottle and preserved with Lugol’s solution (0.05–1.0% by volume depending on cell density). For each sample to be analyzed, a cleaned Utermöhl counting chamber was constructed. Depending on the phytoplankton density of the sample, a settling tower of 5, 10 or 25 mL was used. The tower was secured to the base using a thin film of high vacuum grease. The Lugol’s preserved sample was shaken for 20 seconds to evenly distribute phytoplankton cells, and the appropriate volume added to the settling tower. A cover glass was placed on top of the tower, and the sample was allowed to settle in the dark in a vibration-free location. Minimum settling times were between 17 hrs for 5-mL samples, 34 hrs for 10-mL samples and 74 hrs for 25-mL samples. After settling was complete, the tower was slid from the base of the counting chamber to remove overlying water, and the chamber was covered with a second cover glass.

Enumeration was performed on a Nikon Eclipse TE200 (Southern Microscopes, Inc., Mebane, NC) inverted microscope equipped with phase-contrast optics. One ocular was fitted with a whipple disc and used to define the area of the fields to be counted. Specimens that went beyond the left and bottom edges of the whipple grid were not counted. Before beginning enumeration, the slide was scanned at low power to ensure a relatively uniform cell distribution. Only intact, viable cells were counted. Counts were made as natural units (i.e., cells, filaments, colonies) per a given volume of water. The goal was to count a total of 400–600 cyanobacterial natural units per sample. A minimum of 10 and a maximum of 50 fields were counted at both 400X and 200X. An additional scan of the entire slide was made at 100X to count large or rare taxa, or both. A maximum of 100 fields combined at 200X and 400X was performed on a given sample.

Data were entered into a spreadsheet that calculated natural units/mL. To calculate cells/mL, the average number of cells for colonies and filaments was determined and the units/mL multiplied by these values.

#### Analysis of water samples for microcystins concentrations

To break up cells, water samples were subsampled and sonicated, then filtered and stored at 4°C for toxin analyses. Microcystin analyses were performed within 24–48 hours of receipt of samples. Enzyme linked immunosorbent assay (ELISA) was utilized for the determination of the concentration of total microcystins present. The ELISA assay is based on the polyclonal antibody method described by Chu *et al.* [23] and adapted by An and Carmichael [24]. Antibody-coated plates, standards, and all reagents were supplied by Abraxis LLC (Warminster, PA). The LOD for the total of all microcystin variants using this method was approximately 0.15 μg/L. Microcystins were quantified using a Stat Fax 303+ spectrophotometer (Envirologix, Portland, ME) at a wavelength of 450 nm in conjunction with a reference wavelength of 630 nm. A final estimate for microcystins content was obtained and calculated as the mean of at least two subsamples (i.e., two replicates per subsample).

#### High-volume air samplers

To collect large quantities of material for microcystin analysis, two portable high-volume air samplers (Model TE 5200, Tisch Environmental, Inc., Cleves, OH) were placed on a boat. One sampler collected airborne particles in one filter substrate for total aerosol concentration at a flow rate of 1.5 m^3^/min. For total concentration as well as for particle size distribution at a flow rate of 1.2 m^3^/min, the second sampler housed a five-stage, high-volume cascade impactor (Model SA235, Andersen Instruments, Smyrna, Georgia). Cellulous filters (Filter Paper 41, Whatman International Ltd., Maidstone, UK) were used for stage substrates and backup collection of the samplers. The boat remained stationary, near where water samples were taken. The sampling time usually started between 8 to 8:30 a.m. and ended between 3:30 to 4:30 p.m.

#### Personal Samplers

Personal exposure was measured using a personal sampler connected to a battery-operated pump (SKC, Inc., Eighty Four, PA). If the participant had activities on the boat or beach, the sampler was placed at the lapel near the breathing zone. If participants were in the same recreational group, a single sampler was placed on their boat. A 25-mm cellulose filter (Filter Paper 41, Whatman International Ltd., Kent, UK) was used as the collection substrate. The sampling flow rate was 10.6 L min^−1^ controlled by the sampling pump.

#### Analysis of Air Samples

The analysis of air samples is described by Cheng *et al.* [25]. Briefly, after collection, the high-volume impactor substrates were stored at −20º C. Filters were removed from cold storage and before extraction, equilibrated to room temperature. The whole filter of a personal sampler or a piece of 3.0 cm^2^ cut from the high-volume sampler substrate was placed in a glass vial, and 1 mL of phosphate-buffered saline was added. The vial was placed on a circular rotator (Roto-Torque^®^ Low Speed #10; Cole-Parmer Instruments, Vernon Hills, IL) for 10 min. After rotation, the filter was removed from the extraction tube and the extract was used for ELISA (ELISA kits for microcystin-LR, Production #520011; Abraxis LLC, Warminster, PA) analysis of microcystin. Before analysis—to account for any solution absorbed by the filter—the final volume of the remaining extraction solution was measured. The limit of detection of the microcystin using the ELISA assay was 70 ng/L. For total particle high-volume filter samplers, this corresponded to about 0.0037 ng/m^3^.

#### Waterborne pathogens

We collected 24, 10-L lake water samples at the same locations and times as the samples for algal taxonomy and toxin analyses and concentrated the samples for molecular analysis. Each sample was concentrated using an ultrafiltration procedure based on Hill *et al.* [26]. Ultrafiltration concentrates (~ 400 mL each) were shipped overnight in chilled coolers to the laboratory at the Centers for Disease Control and Prevention (CDC) in Atlanta. We further concentrated viruses using polyethylene glycol (PEG) precipitation (addition of 8% PEG 8000, 0.3M NaCl, and 1% Bovine Serum Albumin; incubation overnight at 4ºC). Final PEG concentrates were ~ 5 mL in volume. DNA and RNA were simultaneously extracted from a volume of 500 μL from each sample [27]. Final nucleic acid extracts were ~100 μL, and 20-μL volumes of nucleic acid extract were used for each PCR and RT-PCR assay. Each sample was assayed for adenoviruses using a broadly reactive real-time PCR assay [28] and a pan-enterovirus real-time RT-PCR assay [29]. PCR inhibition was evaluated in each of the 24 samples by seeding a reagent-grade water control and an aliquot of DNA extract from each sample with the same amount of DNA from a stock of adenovirus 40, and testing these seeded specimens and controls using the adenovirus real-time PCR assay. Differences in CT values between controls and water samples were used as indications of the magnitude of PCR inhibition of the adenovirus assay. This approach to evaluating PCR inhibition for water samples has been previously reported [27].

#### Study participants

During August 4–6, 2006, we recruited study participants on-site. Specifically, we approached approximately 133 people who came to the study lakes for recreational activities. Of these, approximately 29 (22%) refused for various reasons, including that they were either too busy (13), not interested (4), afraid of giving a blood sample (4), or not planning to swim in the lake (5). The remaining three people did not report why they were not interested. Interested individuals read and signed a consent form. They were interviewed on the day of the study in person and later by telephone. After their recreational activities, each provided a blood sample that was analyzed for microcystin concentrations.

### Questionnaires

We conducted personal interviews to collect demographic data (e.g., age, sex, race) and self-reported symptom data including flu-like symptoms (fever, chills, sort throat, headache, earache), eye irritation (blurred vision, eye irritation, red eyes, conjunctivitis), dermatologic symptoms (itchy skin, red skin, hives, skin irritation, rash, infections), respiratory symptoms (cough, shortness of breath, congestion, throat irritation), neurologic symptoms (agitation, confusion, dizziness, lethargy, weakness, seizure, numbness, tremor), and gastrointestinal symptoms (abdominal pain, nausea, vomiting, diarrhea).

We asked whether participants had any of the symptoms during the week before the study as well as before and after recreational activities on the lakes. Using a telephone interview, approximately 10–14 days after exposure we again collected self-reported symptom data.

We used SAS/STAT software Version 9.1 (SAS Institute, Cary, North Carolina) for statistical analyses. For each participant in each time period individual symptoms were summed and then dichotomized to represent any-versus-no symptoms. We used Fisher’s Exact Test [30] to examine whether people in the exposed group reported more frequently having any symptoms than did those in the control group.

#### Blood sample analysis

Methods and parameters used in this study for analysis of microcystins in human blood samples were based on two human exposure episodes in Brazil—one in 1996 and the more recent in 2004 [5, 6, 31, 32, 33]. While both of these events occurred because of contaminated drinking water used in a kidney dialysis facility, the analysis methods are relevant to the current study where exposure resulted from swallowing water or inhaling spray during water-based recreational activities.

Blood samples were collected in 6-ml Vacutainer® tubes (Becton, Dickenson, and Company, Franklin Lakes, New Jersey) containing sodium heparin and then centrifuged. The plasma samples were shipped frozen and stored at −70° C. Blood plasma (0.5 ml) was extracted in 10 ml of methanol. The extract was centrifuged at 7,500 rpm for 15 minutes. The supernatant was transferred into a flask and the pellet was re-extracted in 5-ml methanol and re-centrifuged. The supernatants were combined, and 30 ml of hexane was added. The flask was shaken and the hexane layer removed. The methanol extract was dried down and reconstituted in 6 mL of 5% methanol in 0.1 M acetic acid. The sample was passed through a Waters Oasis HLB® extraction cartridge (Waters Corporation, Milford, MA). The cartridge was washed with 2 mL of 30% methanol in 0.1 M acetic acid. Microcystins were eluted with 10 mL of methanol. This fraction was saved and dried down. The fraction was reconstituted in 1 mL of 20% MeOH and centrifuged for 10 minutes at 1400 x g. The supernatant was passed through a Microcon® YM-10 (10,000 MW cutoff) (Millipore, Billeria, MA) centrifugal filter. The filtrate was used for analysis.

For the microcystins each sample was analyzed using an Enzyme-Linked Immunosorbent Assay (ELISA). The ELISA method was based upon the original polyclonal antibody method described by Chu *et al.* [1990] and adapted by An and Carmichael [24] and Carmichael and An [34]. The limit of quantification (LOQ) for microcystin on the ELISA plate is 0.175 μg/L. The limit of detection (LOD) for microcystin on the ELISA plate is 0.147 μg/L. Samples were run on a Molecular Devics Corp. (Palo Alto, CA), Vmax kinetic microplate reader.

For the single blood sample that was positive (above the limit of detection for the ELISA) for microcystins, electrospray ionization mass spectrometry (ESI/MS) and MS/MS analyses were determined using a Finnigan Thermo-Quest LCQ Duo benchtop LC/MS/MS. The LCQ Duo MS detector was operated using the positive ESI mode run at a 4.0 kV spray voltage. The signals produced were analyzed using Xcalibur software version 1.1 (ThermoFinnigan, San Jose, CA). Microcystins were separated on a C18 ODS (4 mm x 2 mm i.d.) guard cartridge (Phenomenex, Torrance, CA) and a Pursuit C18 3μ PN 3001 – 100 x 020 column (Varian, Palo Alto, California) at a flow rate of 0.2 mL/min. The mobile phase was (A) HPLC grade water containing 0.1 M HOAc and (B) acetonitrle containing 0.1 M HOAc. For microcystin-LR, the mass spectrometer was operated in the full scan mode for MS/MS/-mlz 270–1050. For microcystin-RR, the mass spectrometer was operated in the full scan mode for MS/MS-mlz 285–1080. For microcystin-YR, the mass spectrometer was operated in the full scan mode for MS/MS-mlz 285–1100. The limits of detection and quantification for microcystin-LR, -RR, and -YR by LC/MS without concentration are 0.1, 1, and 10 μg/L, respectively.

In summary, we collected usable blood samples from 96 exposed and 6 unexposed participants. Of the 102 blood samples, 101 samples tested below the limit of detection according to the limit of detection described in the Envirologix Kit (0.147 μg/L). Only one blood sample showed detectable levels of microcystins—about 1.0 μg/L. LC/MS analysis of this sample showed the absence of microcystin-LR, -RR, and -YR. Given the LC/MS results, we concluded that this sample represents either a false positive or exposure from microcystin(s) not identified as microcystin-LR, -RR, or -YR.

## Figures and Tables

**Table 1:** Microcystin concentrations (average of am and pm samples), water quality parameters (average of am and pm samples), and weather parameters measured during the study.

	Station 1			Station 2			Station 3			Station 4		
Water quality parameter (units)	Day 1	Day 2	Day 3	Day 1	Day 2	Day 3	Day 1	Day 2	Day 3	Day 1	Day 2	Day 3
Microcystin concentratio n (μg/L)	2	2	3.5	2	4	3.5	2	2.5	2	2.5	2	3
Water temperature (°C)	28.9	27.2	26.8	28.7	27.7	27.1	28.2	27.3	26.5	27.7	27.9	27.2
pH	8.9	9.0	8.9	8.9	8.8	8.8	8.8	8.5	8.5	9.0	9.0	9.0
Dissolved oxygen (mg/L)	8.7	9.2	9.0	8.8	8.2	7.8	9.7	8.1	7.2	10.2	9.72	9.9
Salinity (ppt)	0.17	0.18	0.18	0.17	0.18	0.18	0.17	0.17	0.18	0.18	0.18	0.18
Turbidity (NTU)	12.4	18.0	0.1	16.0	17.4	3.2	22.4	11.2	0.2	28.4	1.6	21.3*
Sample site depth (m)	2.5	2.4	2.3	2.5	2.8	2.8	1.2	1.2	1.3	0.4	0.4	0.4
Chlorophyll (μg/L)	14.0	15.2	18.2	16.2	16.4	20.2	15.3	17.4	19.5	19.2	17.3	16.8
Total dissolved solids (mg/L)	0.2495	0.2529	0.2482	0.2500	0.2448	0.2474	0.2462	0.2438	0.2466	0.2479	0.2432	0.2454
***Weather***												
Air temperature (°C)	28.8	28.4	27.8	29.2	29.4	28.0	29.3	29.2	24.7	30.0	28.2	25.7
Cloud cover (%)	7.5	55	100	0	60	95	2.5	80	90	10	88	65
Wind speed (mph)	3.6	6.4	2.0	5.2	5.2	6.8	4.0	3.0	8.2	6.0	2.2	6.0
Wind direction (0 – 360°)	315	172	232	175	160	210	298	150	225	180	200	248

* Only the afternoon measurement was available.

**Table 2:** Densities of potentially toxigenic cyanobacteria in water samples from four sampling stations on each of 3 days in the study lake with the algal bloom.

Potentially Toxigenic Cyanobacteria (PTOX) (cells/mL)		Station 1			Station 2			Station 3			Station 4		
Genera	Species	Day 1	Day 2	Day 3	Day 1	Day 2	Day 3	Day 1	Day 2	Day 3	Day 1	Day 2	Day 3
*Anabaena*	*Macrospore*	4,925	5,746	11,492	1,437	10,056	7,183	1,437	456	8,619	7,183	2,873	17,239
	*Spiroides*	2,062	3,932	983	983	2,949	2,873	3,932	286	2,949	3,392	2,949	7,863
	*Sp.*	5,444	6,351	12,702	3,969	11,432	3,811	6,351	2,722	1,684	3,176	7,621	6,447
*Aphanizomenon*	*Aphanizomenonoides/gracile*	25,923	24,295	18,146	24,195	25,404	39,921	18,146	2,592	25,518	25,404	25,404	30,243
	*Issatschenkoi*	7,777	3.024	3,024	2,268	3,629	2,268	3,024	756	2,268	3,629	3,629	3,024
	*Cf. flos-aquae*	5,444	3,176	9,527	6,351	7,621	11,432	3,176	2,722	3,176	7,621	7,621	6,351
*Cylindrospermopsis*	*Raciboskii*	0	0	1,361	340	0	3,266	0	1,167	1,276	0	0	6,850
*Microcystis*	*Cf. botrys*	20,220	5,897	15,726	5,897	18,872	37,744	7,863	6,740	22,115	7,863	7,863	29,487
	*Wesenbergii*	3,213	1,870	3,213	12,853	2,210	6,427	1,190	85	3,213	1,530	1,530	6,427
	*Sp.*	6,049	240	6,049	240	80	29,033	80	480	51,414	0	3,024	9,073
***Total PTOX cells/mL***		81,057	54,431	82,223	58,533	82,253	143,958	45,199	18,006	122,232	61,517	62,514	122,959

**Table 3:** Mass and air concentrations of microcystins collected on each study day using personal samplers carried by study participants or placed on their boats.

Study Day	Mean Sampling Time (min)	Mass of microcystins on filter (ng)			Air concentration of microcystins (ng/m^3^)		
		Mean	Standard Deviation	Range	Mean	Standard Deviation	Range
1	88.9	0.07	0.07	0.006–0.223	0.08	0.09	0.005–0.319
2	134	0.06	0.09	0.017–0.29	0.07	0.014	0.020–0.456
3	NA	<LOD	NA	<LOD	<LOD	NA	<LOD

Note: the air pump flow rate was 0.0106 m^3^/min.

**Table 4:** Self-reported symptom data from study participants engaged in recreational activities in a lake with an algal bloom and a lake without a bloom.

	Study participants doing recreational activities in the lake with the algal bloom				Study participants doing recreational activities in the lake without an algal bloom			
Symptom	7 days before study	Before study activities	After study activities	7–10 days after study activities	7 days before study	Before study activities	After study activities	7–10 days after study activities
*Respiratory symptoms*								
Sore throat	7 (7)	4 (4)	3 (3)	8 (8)	2 (29)	0	0	1 (14)
Congestion	19 (20)	9 (9)	10 (10)	23 (24)	2 (29)	0	0	1 (14)
Cough	5 (5)	4 (4)	1 (1)	5 (5)	1 (14)	0	0	1 (14)
Throat irritation	9 (9)	6 (6)	3 (3)	7 (7)	1 (14)	0	0	1 (14)
Eye irritation	2 (2)	1 (1)	1 (1)	4 (4)	0	0	0	1 (14)
Other respiratory complaint	6 (6)	4 (4)	1 (1)	1 (1)	0	0	0	1 (14)
*Dermatologic symptoms*								
Itchy skin	10 (10)	6 (6)	8 (8)	11 (12)	1 (14)	1 (14)	0	2 (29)
Red skin	7 (7)	6 (6)	2 (2)	2 (2)	2 (29)	1 (14)	0	0
Hives	3 (3)	2 (2)	0	2 (2)	0	0	0	0
Skin irritation	3 (3)	1 (1)	1 (1)	2 (2)	2 (29)	1 (14)	0	0
Rash	5 (5)	4 (4)	2 (2)	5 (5)	2 (29)	1 (14)	0	0
Other skin problem	4 (4)	4 (4)	3 (3)	2 (2)	1 (14)	1 (14)	0	0
*Other symptoms*								
Earache	7 (7)	3 (3)	2 (2)	4 (4)	0	0	0	0
Agitation	3 (3)	3 (3)	0	1 (1)	0	0	0	0
Headache	20 (21)	6 (6)	5 (5)	9 (9)	4 (57)	0	0	1 (14)
Abdominal pain	3 (3)	1 (1)	0	2 (2)	0	0	0	1 (14)
Diarrhea	3 (3)	1 (1)	1 (1)	3 (3)	0	0	0	0

Note: The data are from questionnaires administered for the week before study activities, immediately before and after doing study activities, and during the telephone follow-up interview. Symptoms listed are those reported by more than two study participants in any of the four questionnaires. Values are number of study participants reporting the symptom (%).
